# HIF1α-dependent mitophagy facilitates cardiomyoblast differentiation

**DOI:** 10.15698/cst2020.05.220

**Published:** 2020-03-04

**Authors:** Jin-Feng Zhao, Catherine E. Rodger, George F. G. Allen, Simone Weidlich, Ian G. Ganley

**Affiliations:** 1 MRC Protein Phosphorylation and Ubiquitylation Unit, School of Life Sciences, University of Dundee, Dundee, DD1 5EH, UK.; # Equal contribution.

**Keywords:** mitophagy, HIF1α, NIX, BNIP3, iron chelation, cardiomyocyte, differentiation

## Abstract

Mitophagy is thought to play a key role in eliminating damaged mitochondria, with diseases such as cancer and neurodegeneration exhibiting defects in this process. Mitophagy is also involved in cell differentiation and maturation, potentially through modulating mitochondrial metabolic reprogramming. Here we examined mitophagy that is induced upon iron chelation and found that the transcriptional activity of HIF1α, in part through upregulation of BNIP3 and NIX, is an essential mediator of this pathway in SH-SY5Y cells. In contrast, HIF1α is dispensable for mitophagy occurring upon mitochondrial depolarisation. To examine the role of this pathway in a metabolic reprogramming and differentiation context, we utilised the H9c2 cell line model of cardiomyocyte maturation. During differentiation of these cardiomyoblasts, mitophagy increased and required HIF1α-dependent upregulation of NIX. Though HIF1α was essential for expression of key cardiomyocyte markers, mitophagy was not directly required. However, enhancing mitophagy through NIX overexpression, accelerated marker gene expression. Taken together, our findings provide a molecular link between mitophagy signalling and cardiomyocyte differentiation and suggest that although mitophagy may not be essential *per se*, it plays a critical role in maintaining mitochondrial integrity during this energy demanding process.

## INTRODUCTION

(Macro)Autophagy is a membrane-driven degradation pathway involving the sequestration of cellular material within autophagosomes and concomitant delivery to lysosomes for recycling [1–3]. Under certain circumstances, the autophagy machinery can be engaged to specifically target and eliminate mitochondria in a process termed mitophagy. Mitophagy is thought to play a critical mitochondrial quality-control role by eliminating impaired and/or damaged mitochondria in order to maintain a functional mitochondrial pool, additionally, it may also play a role in metabolic remodelling to enable a cell to respond to changing energy demands [4, 5]. Over the past decades, a wealth of evidence has revealed that accumulation of dysfunctional mitochondria is a key risk factor for aging, cardiovascular diseases and neurodegeneration [4, 5]. The most characterised damage-induced mitophagy pathway, at least in terms of mechanism, involves activation of the Parkinson's-related protein kinase PINK1 and ubiquitin E3 ligase Parkin. This pathway is executed upon collapse of mitochondrial membrane potential and leads to a cascade of ubiquitination of outer mitochondrial membrane proteins that signal for autophagosomal engulfment [6]. Nevertheless, as mitochondria are solely responsible for oxygen-dependent energy production, it is reasonable to assume that mito-phagy is also under metabolic control. Indeed, this appears to be the case, with PINK1/Parkin-dependent mitophagy being overridden in conditions that force oxidative phosphorylation (OXPHOS) over glycolysis [7, 8]. There is also strong evidence for metabolic control of mitophagy in yeast, implying this may be a conserved mechanism [9]. We previously identified that loss of cellular iron generates a robust PINK1/Parkin-independent mitophagy response [10]. Intriguingly, this distinct mitophagy pathway was also under metabolic control: as with the PINK1/Parkin pathway, growth of cells in galactose medium to bypass glycolysis and stimulate OXPHOS, abolished mitophagy [10]. This suggests that the metabolic status of the cell acts as a fundamental mitophagy checkpoint. However, the underlying molecular basis of such a checkpoint, or indeed how loss of iron drives mitophagy, is currently unclear.

While being relevant to pathology, mitophagy is also involved in cellular reprogramming [4]. Energy demands change immensely during differentiation, in which cells can switch their metabolic activity from glycolysis to OXPHOS, as it is the case for pluripotent stem cells, skeletal and cardiac muscle cells [11–13]. Compared to cardiac myoblasts, which display a sparse mitochondrial population, the mature cardiomyocyte harbours a complicated mitochondrial network accompanied by increased mitochondrial content and enhanced oxidative metabolism [14, 15]. To remodel the mitochondrial network during development, which is primed for OXPHOS metabolism, removal of immature mitochondria by mitophagy is thought to be necessary. A growing body of research has shown that mitophagy-driven mitochondrial reorganization plays an essential role in cardiac progenitor cell differentiation and perinatal cardiomyocyte maturation [16, 17]. We previously showed that the mouse foetal heart displayed a very low incidence of mitophagy at embryonic day 14.5 (E14.5) but contains extensive areas or patches of cardiomyocytes undergoing very high rates of mitophagy by E17.5 [18]. Although mitophagy has been reported to direct mitochondrial maturation in perinatal hearts, via a PINK1/mitofusin 2 (Mfn2)/Parkin-dependent pathway [16], there is currently no evidence showing how mitophagy contributes to foetal heart development.

In this study, we sought to identify molecular mechanisms behind iron-chelation-induced mitophagy and determine if this pathway is functionally relevant in a physiological setting. In contrast to depolarization-induced mitophagy and PINK1/Parkin activation, mitophagy induction upon iron-chelation, with the compound deferiprone (DFP), required HIF1α-dependent upregulation of BNIP3 and NIX. Using a cell line model of cardiomyocyte differentiation, we found that HIF1α was stabilised during differentiation and induced mitophagy in a NIX-dependent manner. Interestingly, through manipulation of NIX expression levels, we were able to show that mitophagy was not essential for differentiation, but rather has the capacity to influence the rate at which it occurred.

## RESULTS

### HIF1α is essential for iron chelation-induced mitophagy

**Figure 1:**
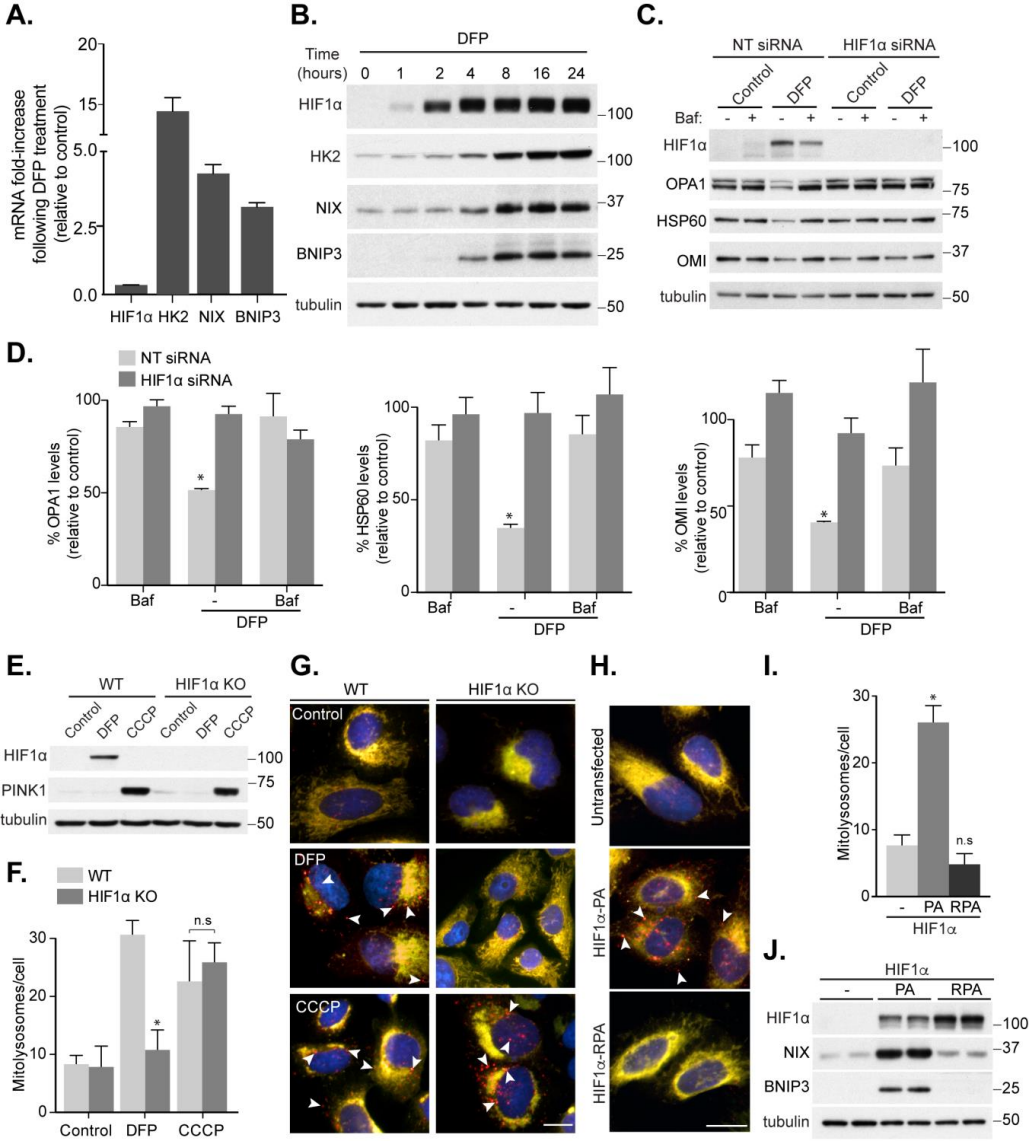
FIGURE 1: HIF1α is essential for mitophagy induced by loss of iron. SH-SY5Y cells were treated with 1 mM DFP for 24 h **(A)** or the indicated length of time **(B)** prior to lysis. **(C)** SH-SY5Y cells were transfected with non-targeting (NT) siRNA or siRNA targeting HIF1α. After 48 h of knockdown, cells were treated with 1 mM DFP for an additional 24 h with/without the addition of 50 nM bafilomycin A1 (Baf A1) for the final 16 h of treatment. **(D)** Quantitation from (C) of mitochondrial proteins relative to control condition. Mitophagy reporter (*mito*-QC) WT or HIF1α KO U2OS cells were treated with 1 mM DFP or 20 μM CCCP for 24 h prior to lysis **(E)** or fixation **(F-G)**. **(F)** Quantitation from (G) of mean mitolysosome (red-only) puncta per cell as indicated. **(H)** Representative images from *mito*-QC U2OS cells in combination with either Flag-HIF1α P402A/P564A (PA) or Flag- HIF1α P402A/P564A/R27G (RPA). **(I)** Quantitation from (H) of mean mitolysosome puncta per cell as indicated. **(J)** Control U2OS cells or U2OS cells stably expressing either Flag-HIF1α-PA or Flag-HIF1α-RPA were lysed and subject to immunoblot analysis. All quantitative data are mean ± SEM from 3 independent experiments. Arrows highlight mitolysosomes. Scale bar, 10 μm. * *P* < 0.05, n.s, not significant.

It is well established that cellular iron depletion can mimic hypoxic conditions through stabilization of the oxygen-sensitive transcription factor HIF1α [19]. We had previously found that various mitophagy-inducing iron chelators led to HIF1α stabilization, though an absolute mitophagic requirement for HIF1α was not tested [10]. To further investigate a potential role for HIF1α, we first confirmed that iron chelation with DFP caused stabilization of active HIF1α by carrying out transcriptional analysis of HIF1α-dependent genes. Following DFP treatment in SH-SY5Y cells, we found a large induction of the known HIF1α target genes Hexokinase 2 and the mitophagy-related *BNIP3L/NIX* and *BNIP3*, both at the mRNA and protein level **(Fig. 1A, B)**, confirming HIF1α activation. Interestingly, the protein levels of these genes are robustly increased after 8 h of DFP treatment, which also correlates with our previously observed increase in mitophagy [10]. If HIF1α is important for mitophagy induction, then hypoxia itself should induce mitophagy. Indeed, we have previously found this to be the case and confirmed those results here using SH-SY5Y cells expressing our well characterized *mito*-QC mitophagy reporter system ([10] and Fig. S1A). Briefly, these cells stably express a tandem mCherry-GFP tag attached to the outer mitochondrial membrane (OMM, via a localization signal derived from the protein FIS1). Under normal cytosolic conditions, both mCherry and GFP (green fluorescent protein) fluoresce and mitochondria appear yellow. However, upon mitophagy, the delivery of mitochondria to acidic lysosomes results in quenching of the GFP signal and the appearance of mCherry-only fluorescence. In line with a role for HIF1α, mitophagy was robustly induced under hypoxic conditions (Fig. S1A). To further implicate HIF1α in iron chelation-induced mitophagy, we first employed small interfering (si)RNA. Treatment of SH-SY5Y with DFP resulted in an approximate 50% loss of multiple mitochondrial proteins over 24 h, which was sensitive to the lysosomal inhibitor bafilomycin A1 (Baf A1) - indicating significant mitophagy **(Fig. 1C, D)**. However, siRNA-mediated depletion of HIF1α abolished this loss. To confirm this, and rule out siRNA off-target effects, we utilised CRISPR/Cas9 to generate HIF1α knock out (KO) cells. Though we were unsuccessful in generating an SH-SY5Y HIF1α KO cell line, we were able to obtain HIF1α KO U2OS cells. KO was confirmed by genomic sequencing (see Methods section) and via western blotting of HIF1α **(Fig. 1E)**. Through our mitophagy reporter, we were able to demonstrate a significant block in DFP-induced mitophagy in these cells compared to wild-type (WT) U2OS **(Fig. 1F, G)**. We next asked if HIF1α is also required for mitophagy in response to carbonyl cyanide m-chlorophenyl hydrazone (CCCP) treatment, a depolarizing agent commonly used as an inducer of PINK1/Parkin-dependent mitophagy [20]. The initial step in this pathway is stabilization of PINK1 on the OMM, followed by the subsequent recruitment and activation of Parkin. In HIF1α KO cells, PINK1 was clearly stabilized after CCCP, but not DFP, treatment **(Fig. 1E)** and mitophagy proceeded normally **(Fig. 1F, G)**. Therefore, in contrast to iron chelation, activation of this pathway is independent of HIF1α.

If HIF1α stabilization is a key initial event during DFP-induced mitophagy, then forced expression of HIF1α, under normal growth conditions, should stimulate mitophagy independently of iron chelation. To test this, we expressed a constitutively stable HIF1α mutant, which contains mutations to the two degradation-targeting proline residues that become hydroxylated during normoxia (P402A/P564A, HIF1α-PA [21]), in our mitophagy reporter U2OS cells. Satisfyingly, expression of the constitutively stable HIF1α alone resulted in significant mitophagy induction (**Fig. 1H-J**). Additionally, this was dependent on HIF1α transcriptional activity as a constitutively stable but DNA-binding deficient HIF1α mutant (P402A/P564A/R27G, HIF1α-RPA [22]) failed to trigger mitophagy, despite similar levels of expression **(Fig. 1H-J**). Taken together, these results demonstrate the distinct requirements for specific forms of autophagy, with HIF1α being important for mitophagy in response to iron chelation.

### NIX and BNIP3 are critical mediators of HIF1α-dependent mitophagy

**Figure 2:**
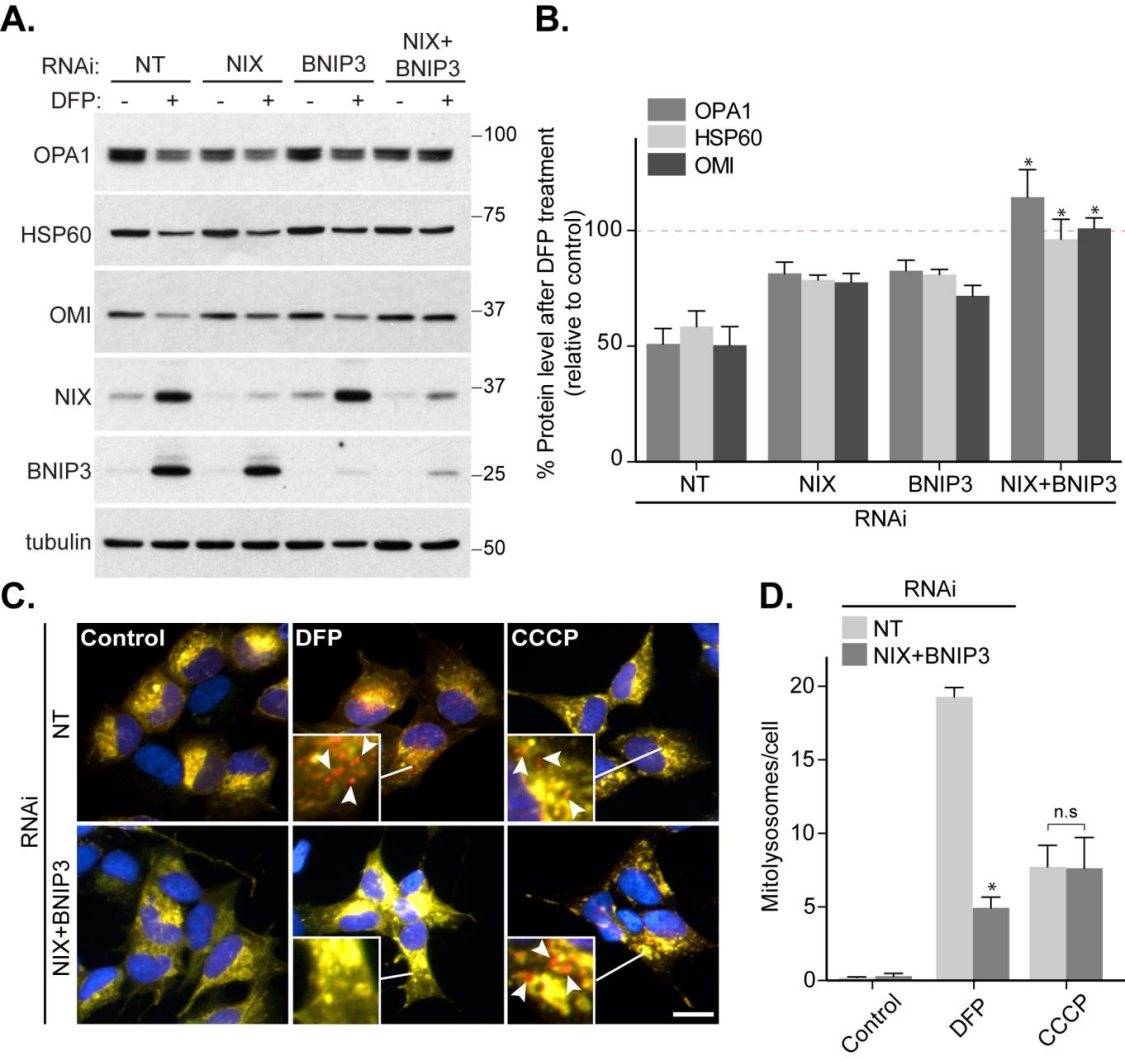
FIGURE 2: NIX and BNIP3 redundancy in DFP-induced mitophagy. **(A)** Control SH-SY5Y cells or SH-SY5Y cells stably expressing BNIP3 shRNA were either transfected with non-targeting (NT) siRNA or NIX siRNA. 48 h post-transfection cells were treated with 1 mM DFP for a further 24 h. **(B)** Quantitation from (A) of OPA1, HSP60 and OMI levels relative to control condition. Dotted line represents control value (100%). **(C)** Control SH-SY5Y cells were transfected with NT siRNA and SH-SY5Y cells stably expressing BNIP3 shRNA were transfected with an equal amount of NIX siRNA. Cells were treated with 1 mM DFP or 20 μM CCCP for 24 h. Arrows highlight examples of mitolysosomes. Scale bar, 10 μm. **(D)** Quantitation from (C) of mitolysosomes per cell as indicated. All quantitative data are mean ± SEM from 3 independent experiments. * *P* < 0.05, n.s, not significant.

The mitophagic requirement for HIF1α transcriptional activity implies that upregulation of downstream proteins mediate mitophagy. Two HIF1α-regulated OMM-anchored proteins, BNIP3 and NIX, have previously been linked to mitophagy as they bind to autophagic ATG8s through conserved LC3-interacting regions (LIRs) [23–27]. Indeed, we do see that these two proteins are upregulated upon DFP treatment **(Fig. 1A, B)**. In our previous work, we found that depletion of BNIP3 did not significantly affect DFP-induced mitophagy [10] and here we extended the study to include analysis of NIX. RNAi-mediated depletion of BNIP3 or NIX alone in SH-SY5Y cells did not impede mitophagy **(Fig. 2A, B)**, consistent with our previous data. In contrast, combined depletion of both BNIP3 and NIX completely abolished the DFP-induced loss of multiple mitochondrial proteins, including OPA1, HSP60 and OMI **(Fig. 2A, B)**. This suggests that both these proteins can regulate mitophagy but in SH-SY5Y cells a degree of redundancy exists between them, which is perhaps unsurprising given their high degree of sequence similarity. It is possible that BNIP3 and NIX perform a similar role in marking mitochondria for mitophagy as ubiquitylation of OMM proteins does during depolarization-induced mitophagy, thus explaining the dispensable need for PINK1 and Parkin. In support of this, and consistent with our HIF1α data **(Fig. 1E-G)**, our mitophagy reporter assay showed that while BNIP3 and NIX were essential for DFP-induced mitophagy, their loss did not block CCCP-induced mitophagy **(Fig. 2C, D)**.

mTOR (mammalian target of rapamycin) inhibition is a key requirement for autophagosome formation under starvation conditions and is perhaps the best characterised mode of autophagy initiation [28]. Given this, we analysed mTOR activity in cell lysates following DFP-induced mitophagy. We found that amino acid starvation, with Earls Balanced Salt Solution, resulted in the expected robust dephosphorylation of the autophagy-initiating kinase ULK1 at serine 757 (mTOR site [29, 30]), but in contrast DFP treatment did not (Fig. S1A, B). This implies mTOR inhibition may not be a key mediator of mitophagy under these conditions. This was further supported by the fact that DFP-induced mitophagy was not influenced when combined with pharmacological mTOR inhibition using AZD8055, a highly specific ATP-competitive inhibitor [31] (Fig. S1C, D). Thus, the role of ULK1 here, and its mechanism of activation, remains unclear and will be a focus of future endeavours.

### Mitophagy is induced during cardiomyocyte differentiation

We had previously found that mouse foetal hearts increased their degree of mitophagy prior to birth [18]. Further analysis of this demonstrates that mitophagy peaks at day E17.5 (Fig. S2). Interestingly, this increase in mitophagy is not uniform and appears as areas, or patches, of high cellular mitophagy during this period, before changing to a more uniform and lower level following birth. This implies cardiac mitophagy may play a key developmental role, and indeed, mitophagy has previously been reported to be involved in the development of various tissues, including heart [16, 17, 32–34]. To further understand the mechanistic basis and physiological significance of mitophagy during embryonic heart development, we used the H9c2 cardiomyoblast cell line as an experimentally tractable model, which displays many similarities to primary cardiomyocytes [35].

**Figure 3:**
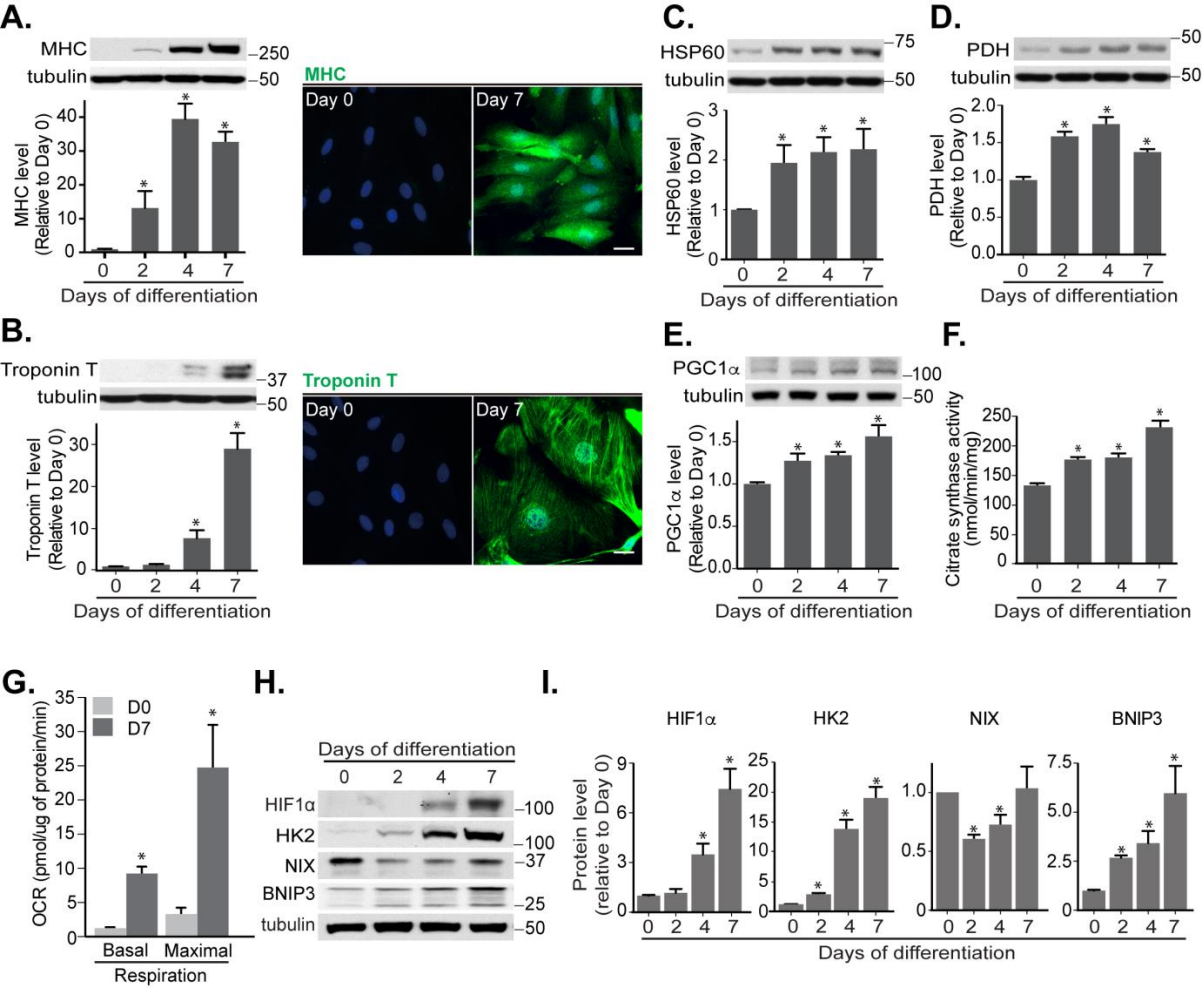
FIGURE 3: Mitochondrial functions increase during cardiomyoblast differentiation. Protein levels and immunofluorescence staining of myosin heavy chain (MHC) **(A)** and cardiac Troponin T **(B)** were performed in H9c2 cells following differentiation for 0, 2, 4 and 7 days. HSP60 **(C)**, PDH **(D)**, PGC1α **(E)** protein levels and citrate synthase activity **(F)** were determined during this period. **(G)** Oxygen consumption rate (OCR) was measured in H9c2 cells following 7 days differentiation. **(H-I)** HIF1α, Hexokinase 2 (HK2), NIX and BNIP3 protein levels were examined and quantified in H9c2 cells during the indicated days of differentiation. α-tubulin was used as a loading control. All quantitative data are mean ± SEM from 3 independent experiments. Scale bar, 20 μm. * *P* < 0.05.

In the presence of low serum and retinoic acid, H9c2 cells are known to differentiate over a period of 7 days and during this process they express mature cardiomyocyte markers, including myosin heavy chain (MHC) and cardiac Troponin T. We confirmed that this was the case in our experimental set-up and observed increased MHC and Troponin T at both the protein **(Fig. 3A, B)** and mRNA level (Fig. S3A). Also, mitochondrial mass increased, likely caused by mitochondrial biogenesis, as evidenced by enhanced protein levels of mitochondrial HSP60, PDH, PGC1α and citrate synthase activity **(Fig. 3C-F)**. We further examined the oxygen consumption rate (OCR), which represents mitochondrial metabolic activity, in cardiomyoblasts and cardiomyocytes. Compared to undifferentiated myoblast cells, mature cardiomyocytes dramatically increased their OCR, indicating an increased OXPHOS capacity, consistent with their increased mitochondrial content **(Fig. 3G)**.

To understand whether HIF1α-mediated mitophagy occurs during cardiomyoblast differentiation, we looked for changes in levels of HIF1α and its downstream proteins. Indeed, HIF1α and BNIP3 protein and mRNA levels were increased during differentiation (Fig. S3B, C and H, I). Surprisingly, NIX protein and mRNA expression were reduced during the first 4 days of differentiation but then recovered in-line with mitophagy induction during the latter stages of cardiomyocyte differentiation (Fig. S3D and H, I).

**Figure 4:**
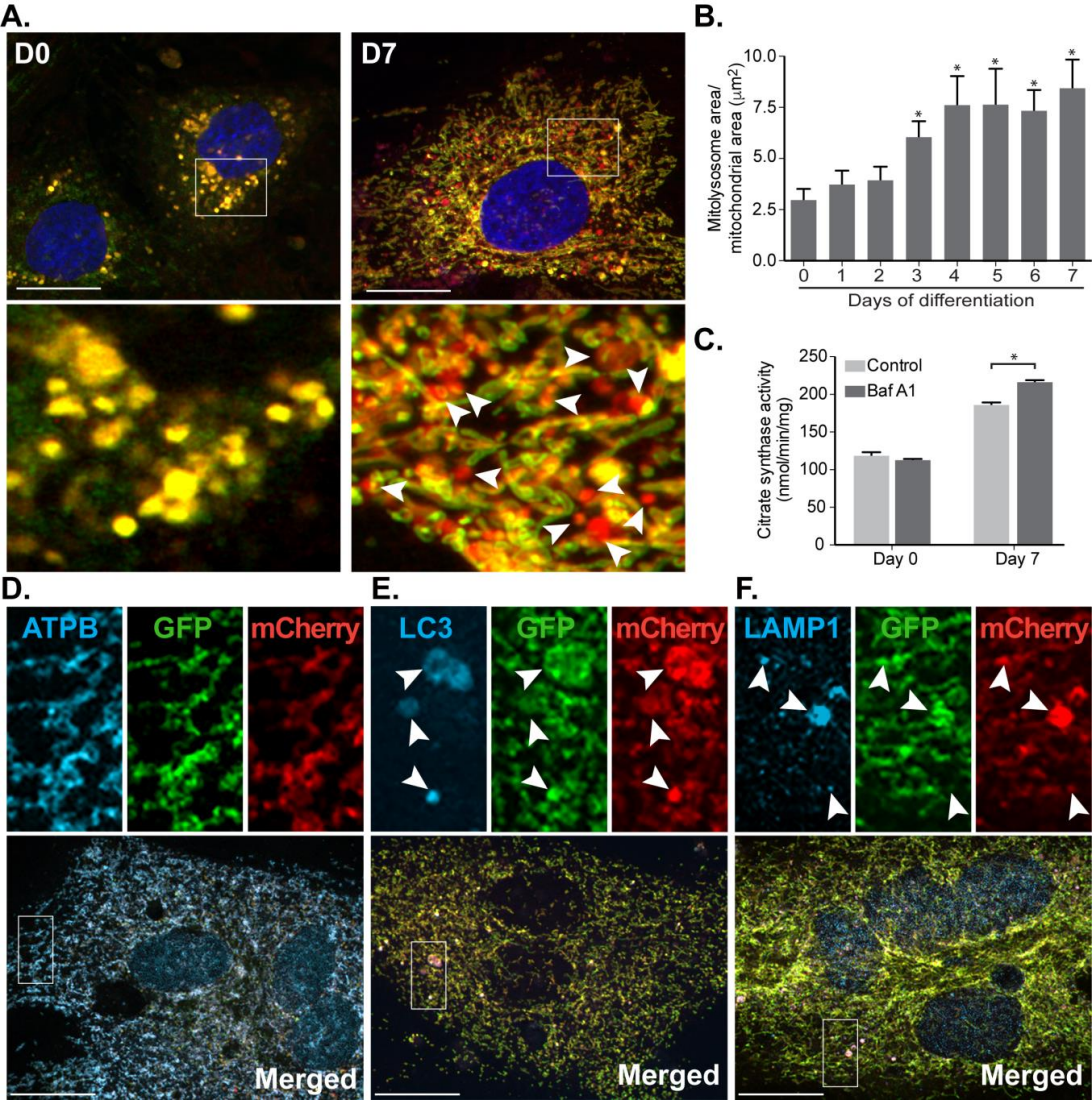
FIGURE 4: Mitophagy increases progressively during cardiomyocyte differentiation. **(A)** Representative images from *mito*-QC reporter H9c2 cells during differentiation. **(B)** Quantitation of total mitolysosome area per mitochondrial content was analysed during cardiomyocyte differentiation. **(C)** H9c2 cells were cultured in differentiation medium for 7 days and 50 nM Baf A1 was added into medium for the last 16 h prior to lysis. Immunostaining of beta subunit of ATP synthase (ATPB) **(D)**, LC3 **(E)** and LAMP1 **(F)** were performed in H9c2 cells differentiated for 7 days prior to fixation. Arrows indicate structures positive for both red-puncta and LC3 or LAMP1. Scale bar, 20 μm. All quantitative data are mean ± SEM from 3 independent experiments. * *P* < 0.05.

Given the increased levels of HIF1α, we next examined mitophagy by expressing the *mito*-QC reporter in the H9c2 cells. Upon differentiation, mitophagy is progressively induced, with levels peaking after 4 days **(Fig. 4A, B)**. As expected, the *mito*-QC reporter was mitochondrial, as evidenced by extensive co-localisation with ATP synthase **(Fig. 4D)** and the red-only puncta were indicative of mitophagy given their co-localisation with LC3 and LAMP1 **(Fig. 4E** and **F)**. We do note that in these co-localization experiments, some GFP fluorescence is retained in lysosomal structures. This is due to the fact that immunostaining requires cell membrane permeabilization, which results in dequenching of the GFP signal. Regardless, these experiments confirm correct localization of the reporter in H9c2 cells. As an alternative, *mito*-QC-independent method to monitor mitophagy, we examined mitochondrial mass via measurement of mitochondrial citrate synthase activity. Given the presumably large increase in mitochondrial biogenesis relative to turnover, the decrease in mitochondrial mass due to mitophagy was hard to detect. Nevertheless, we saw a significant lysosomal-sensitive change in citrate synthase activity following differentiation, strongly supporting the notion of increased mitophagy **(Fig. 4C)**.

### HIF1α is required for mitochondrial remodelling and mitophagy during cardiomyocyte differentiation

**Figure 5:**
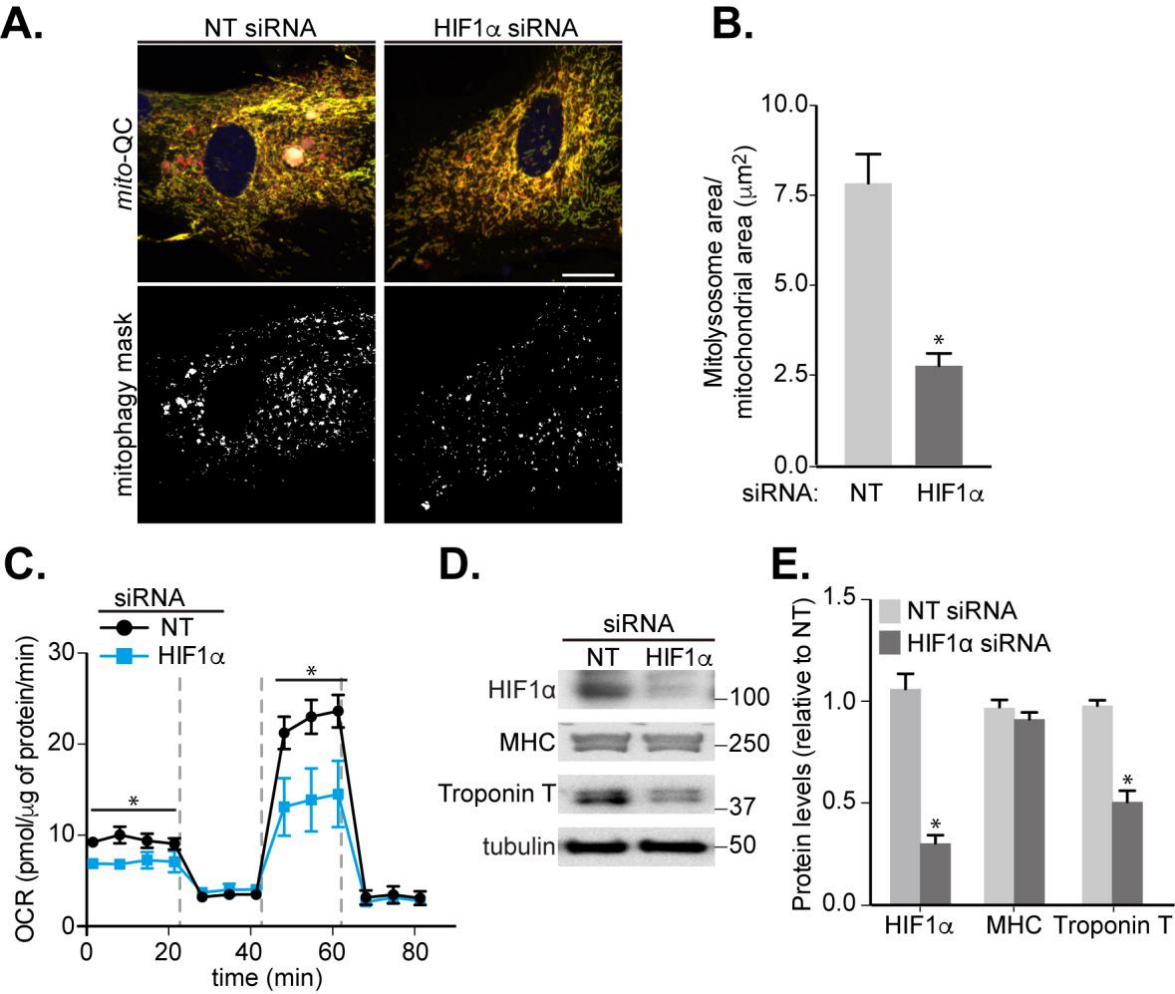
FIGURE 5: Involvement of HIF1α in mitophagy and cardiomyocyte differentiation. H9c2 cells were cultured in differentiation medium for 7 days. **(A)** Representative images of *mito*-QC H9c2 cells transfected with non-targeting (NT) siRNA or HIF1α siRNA at day 4 of differentiation. Mitophagy mask represented as the mCherry/GFP ratio intensity above the mean of mCherry intensity. **(B)** Quantitation from (A) of total mitolysosome area per mitochondrial content as indicated. **(C)** OCR was measured following 7 days H9c2 differentiation with cells transfected with NT siRNA or HIF1α siRNA at day 4. 1 μM oligomycin A, 1 μM FCCP and 1/2 μM rotenone/antimycin A were injected at the indicated times to determine the proportion of oxygen consumption due to ATP turnover, maximal rate of respiration and amount of proton leak respectively. **(D-E)** HIF1α, MHC and cardiac Troponin T protein levels were examined in 7 days-differentiated H9c2 cells transfected with NT or HIF1α siRNA at day 4. α-tubulin was used as a loading control. Scale bar, 20 μm. All quantitative data are mean ± SEM from 3 independent experiments. * *P* < 0.05.

Previously published work has indicated that HIF1α is required for skeletal myoblast differentiation [36]. As our results showed that HIF1α is crucial for mitophagy in response to iron chelation **(Fig. 1)**, we further determined whether HIF1α regulates the observed mitophagy during cardiomyoblast differentiation. To examine this, we used siRNA-mediated depletion of HIF1α and applied this at day 4 of differentiation, as this was the period when HIF1α levels and mitophagy were found to increase (**Fig. 3H, I** and **4B**). Here, the *mito*-QC assay demonstrated a significant block in mitophagy in the siHIF1α-treated group compared to non-targeting (NT) group **(Fig. 5A, B)**. Given the large number of mitochondria in these cells, to aid mitophagy visualization in the micrographs, we also displayed the mCherry-only signal as a “mitophagy mask” **(Fig. 5A**, bottom panels). Moreover, knockdown of HIF1α decreased the mitochondrial OCR and cardiomyoblast differentiation, as evidenced by reduced levels of cardiac Troponin T **(Fig. 5C-E)**. Interestingly, depletion of HIF1α did not reduce levels of MHC, suggesting that HIF1α may regulate a specific stage of differentiation, or that MHC is already undergoing increased transcription before siRNA of HIF1α occurs. The latter is possible given that MHC levels rise following two days of differentiation and before Troponin T, which occurs after four days **(Fig. 3A, B)**. To further confirm the significance of HIF1α in cardiomyocyte differentiation, we expressed the previously mentioned constitutively stable HIF1α mutants, HIF1α-PA (constitutively stable HIF1α expression) or HIF1α-RPA (constitutively stable but DNA-binding deficient), in our mitophagy reporter H9c2 cells. As expected, expression of HIF1α-PA increased HIF1-dependent transcription, while HIF1α-RPA did not. The latter also caused a significant block in mitophagy (Fig. S4).

### NIX-dependent mitophagy is dispensable for the initial stages of cardiomyoblast differentiation

**Figure 6:**
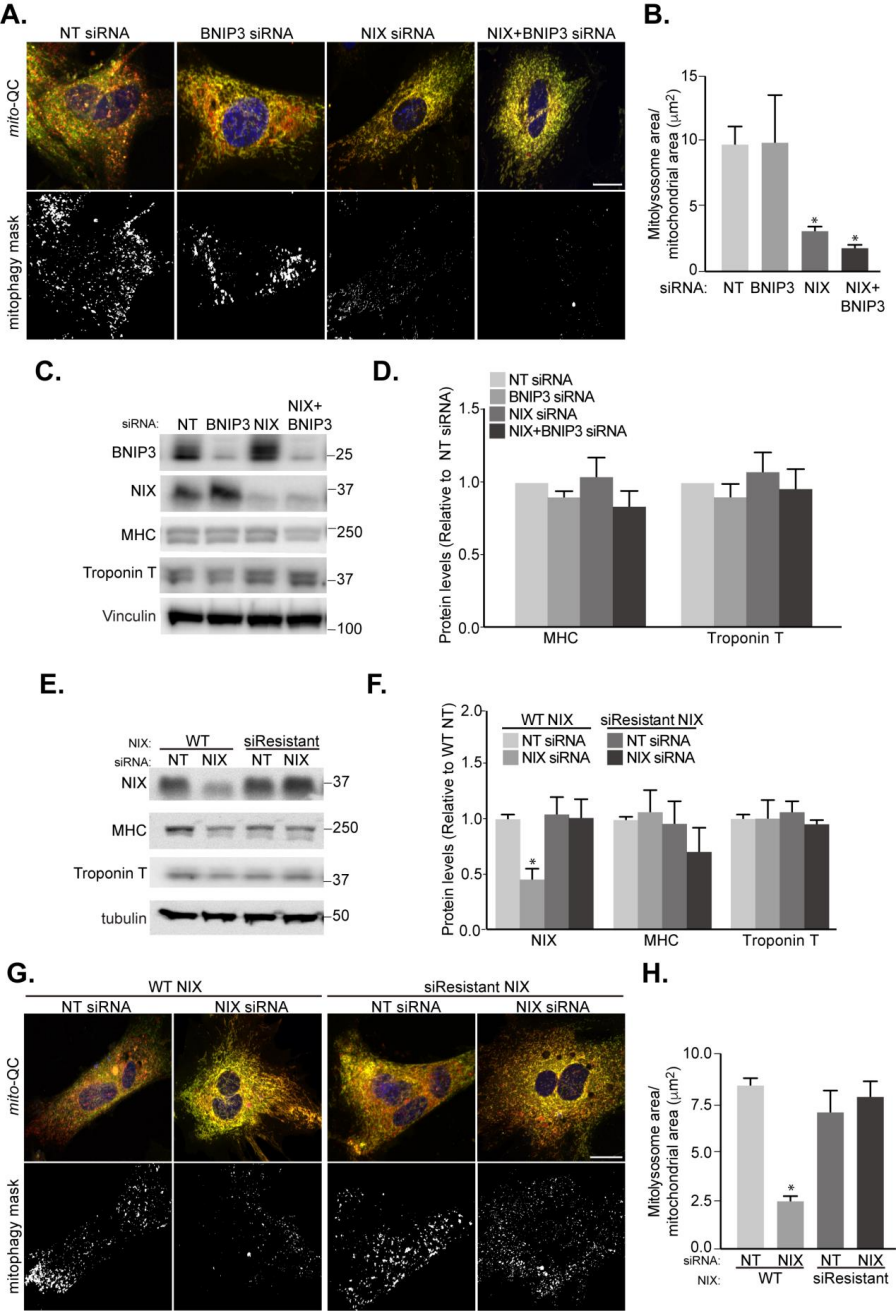
FIGURE 6: NIX-mediated mitophagy is not essential for cardiomyocyte differentiation. H9c2 cells were cultured in differentiation medium for 7 days. **(A)** Representative images of *mito*-QC H9c2 cells transfected with non-targeting (NT), BNIP3, or NIX siRNA as well as NIX and BNIP3 (NIX/BNIP3) siRNA in combination at day 4 differentiation. Mitophagy mask represented as the mCherry/GFP ratio intensity above the mean of mCherry intensity. **(B)** Quantitation from (A) of total mitolysosome area per mitochondrial content as indicated. **(C-D)** BNIP3, NIX, MHC and cardiac Troponin T protein levels were examined after H9c2 cells transfected with NT, BNIP3, NIX or NIX/BNIP3 siRNAs at day 4 differentiation. Vinculin was used as a loading control. **(E)** H9c2 cells stably expressing WT or siRNA resistant NIX were cultured in differentiation medium for 7 days. NT siRNA or NIX siRNA was applied into medium at day 4 differentiation. NIX, MHC and cardiac Troponin T protein levels were examined after 7 days differentiation. Quantitation from (E) of indicated proteins was shown in **(F)**. α-tubulin was used as a loading control. **(G)** Representative images of NIX WT or siRNA-resistant cells transfected with NT siRNA or NIX siRNA at day 4. **(H)** Quantitation from (G) of total mitolysosome area per mitochondrial content as indicated. Scale bar, 20 μm. All quantitative data are mean ± SEM from 3 independent experiments. * *P* < 0.05.

Our findings showed that NIX and BNIP3 were essential for HIF1α mediated mitophagy upon iron chelation in SH-SY5Y cells **(Fig. 2)**. We therefore investigated whether NIX and BNIP3 were required for the mitophagy we observed during H9c2 differentiation, and in turn, if this mitophagy influenced cardiomyocyte differentiation. Interestingly, the patterns of NIX and BNIP3 expression differ during differentiation **(Fig. 3H, I)**, implying they may be under distinct regulation and/or have different roles in H9c2 cells. siRNA-mediated depletion of NIX alone was sufficient to significantly impair mitophagy **(Fig. 6A, B)**. In contrast to SH-SY5Y cells, depletion of BNIP3, either alone or in combination with NIX, did not have an additive effect over NIX siRNA, implying that NIX is the prime regulator of mitophagy dur-ing differentiation **(Fig. 6A, B)**. We do not fully understand why there appears to be more mitophagy redundancy between NIX and BNIP3 in SH-SY5Y cells compared to H9c2 cells, but this could potentially relate to tissue-specific effects and distinct protein interactions, given the very different origins between these cell lines (bone marrow vs. heart). Regardless, loss of mitophagy did not affect the expression of differentiation markers **(Fig. 6C, D)**.

Given the propensity of H9c2 cells to stop dividing and differentiate during clonal selection, we found it challenging to generate CRISPR KOs of our genes of interest in order to confirm the siRNA phenotypes. Therefore, to test the specificity of NIX siRNA in terms of off-target effects, H9c2 cells that stably express WT NIX or an siRNA-resistant form were generated. The levels of expression of both forms of NIX were close to endogenous and depletion was only observed with WT protein upon siRNA treatment **(Fig. 6E-F)**. siRNA of NIX blocked mitophagy in cells expressing WT NIX but failed to do so in cells expressing the siRNA-resistant form, thus confirming that mitophagy inhibition is not due to siRNA off-target effects **(Fig. 6G-H)**. Even though mitophagy was blocked, differentiation in terms of MHC and Troponin T expression, was not impeded **(Fig. 6E, F)**. This is in contrast to loss of HIF1α, which blocked mitophagy as well as differentiation **(Fig. 5A, B** and **D, E)**. Thus, HIF1α likely plays multiple roles during cardiomyocyte differentiation.

**Figure 7:**
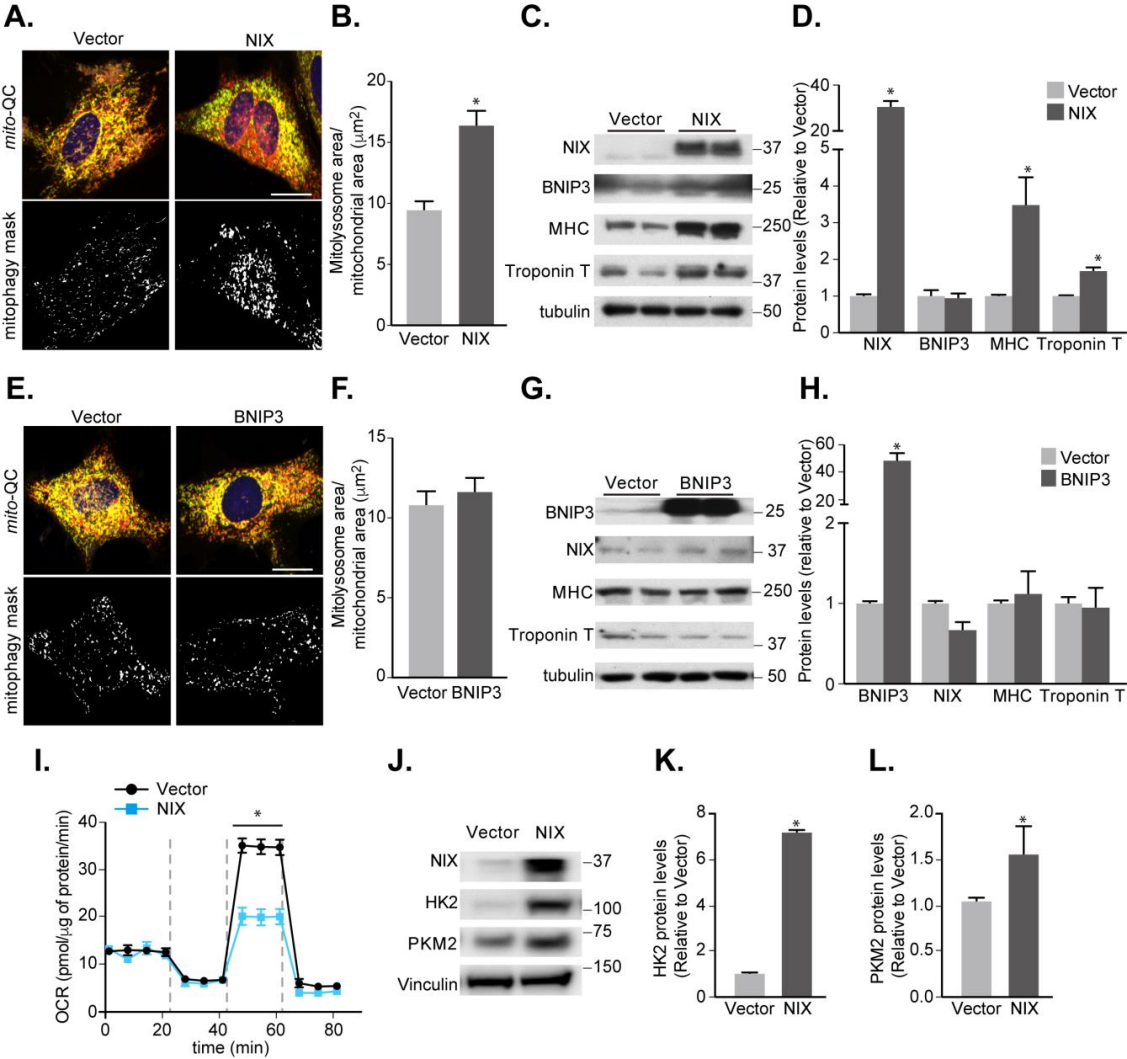
FIGURE 7: Increasing NIX-dependent mitophagy promotes cardiomyocyte differentiation. **(A)** Representative images from *mito*-QC H9c2 vector control cells or cells overexpressing NIX cultured following 7 days differentiation. Mitophagy mask represented as the mCherry/GFP ratio intensity above the mean of mCherry intensity. **(B)** Quantitation from (A) of total mitolysosome area per mitochondrial content as indicated. **(C)** Representative immunoblot of NIX, BNIP3, MHC and cardiac Troponin T protein levels from 7 days differentiated H9c2 cells stably overexpressing NIX. **(D)** Quantitation of data from (C). **(E)** Representative images from *mito*-QC H9c2 cells overexpressing vector or BNIP3 cultured in differentiation medium for 7 days. **(F)** Quantitation from (E) of total mitolysosome area per mitochondrial content as indicated. **(G)** Representative immunoblot of NIX, BNIP3, MHC and cardiac Troponin T protein levels from 7 days differentiated H9c2 cells stably overexpressing BNIP3. **(H)** Quantitation of data from (G). **(I)** Oxygen consumption rate (OCR) was measured after H9c2 cells stably overexpressing vector or NIX following 7 days differentiation. **(J)** Immunoblot of HK2 and Pyruvate kinase (PKM2) from H9c2 cells stably overexpressing vector or NIX cultured in differentiation medium for 7 days. Quanitation of HK2 **(K)** and PKM2 **(L)** is shown. Scale bar, 20 μm. All quantitative data are mean ± SEM from 3 independent experiments. * *P* < 0.05.

The fact that mitophagy is occurring during differentiation suggests it is playing an important role here - in spite of not being absolutely required for key marker gene expression. Thus, it is possible that mitophagy facilitates this mechanism, by keeping metabolic conditions optimal dur-ing this resource-demanding process of differentiation. To investigate this, we examined whether enhancing mitophagy, via overexpression of NIX, could impact differentiation **(Fig. 7)**. A thirty-fold overexpression of NIX during H9c2 differentiation resulted in an almost two-fold increase in mitophagy by day 7 **(Fig. 7A, B)**. Surprisingly, this significantly enhanced expression of both the differentiation marker genes, MHC and Troponin T **(Fig. 7C, D)**. Consistent with a role for mitophagy in this, overexpression of the closely related BNIP3, which did not significantly enhance H9c2 mitophagy, failed to increase marker gene expression **(Fig. 7E-H)**. We also found that NIX overexpression did not increase caspase cleavage (Fig. S5), implying NIX's role in apoptosis is not influencing mitophagy in this instance [37].

How then is enhanced mitophagy driving differentiation? Previous work has shown that mitophagy helps maintain a glycolytic state that is required during differentiation of retinal ganglion cells as well as polarization of M1 macrophages [32]. Thus, we wondered whether enhancing mitophagy is causing a further shift towards a glycolytic state. We found that overexpressing NIX decreased OCR during differentiation in H9c2 cells **(Fig. 7I)**, and in parallel resulted in increased expression of the key rate-limiting glycolytic enzymes, Hexokinase 2 and Pyruvate kinase **(Fig. 7J-L)**. This suggests that enhanced mitophagy does indeed facilitate metabolic reprogramming towards a glycolytic state during cardiomyocyte differentiation, in-line with the other previously mentioned cell types [32]. We have found that Hexokinase 2 is normally upregulated during mitophagy induction (**Fig. 1A, B** and **3H-I**), but how NIX overexpression further enhances this is currently unclear. Though the exact mechanism of how mitophagy upregulates glycolysis remains to be determined, this work suggests that mitophagy-driven metabolic reprogramming is a general phenomenon during differentiation.

## DISCUSSION

We previously found that DFP stimulated mitophagy in a manner that was distinct from the PINK1/Parkin pathway [10]. To gain insight into the mechanism, we demonstrated in SH-SY5Y cells that DFP-induced mitophagy required HIF1α stabilization as well as its transcriptional activity. This HIF1α-dependent transcription was responsible for upregulation of the mitophagy receptor proteins BNIP3 and NIX. Interestingly, there appeared to be a degree of redundancy between these proteins, as loss of both was required to fully block mitophagy in SH-SY5Y cells. Given the high degree of similarity between these proteins, such a level of redundancy is perhaps not surprising.

To examine this mitophagy pathway in a more physiological setting, we moved to a cardiomyocyte differentiation model using H9c2 cells. This was guided by our earlier data indicating mitophagy occurred in developing mouse hearts ([18] and Fig. S2), as well as previously published work in the neonate mouse heart [16] and during differentiation of C2C12 myoblasts [33]. Here, we found that during differentiation HIF1α was upregulated and was required for cardiomyoblast differentiation as well as mitophagy. In contrast to SH-SY5Y cells, levels of BNIP3 and NIX did not display the same expression pattern. Unlike BNIP3, NIX was already expressed in myoblasts and upon differentiation its levels decreased initially before being restored, in-line with the increase in mitophagy. We do not know the reasons for the discrepancy between NIX and BNIP3 expression levels, but interestingly in the heart, NIX can also be upregulated through G-Protein coupled receptor signalling [38], which may account for the differences observed here. Though we were able to show that forced expression of HIF1α increased NIX protein levels (Fig. S4C, D), the results suggest multiple transcription networks may be at play here and further work is needed to determine this. Also, unlike the SH-SY5Y cells, depletion of NIX alone was sufficient to cause a significant block in mitophagy. Intriguingly, this is consistent with published *in vivo* data [39]. In mice lacking NIX, but not BNIP3, cardiac enlargement with decreased left ventricular dilation was observed at 60 weeks. However, in mouse hearts that lacked both NIX and BNIP3, this phenotype was observed much earlier at 30 weeks. Additionally, these hearts contained a striking increase in mitochondrial content, consistent with a block in mitophagy [39]. Thus, in heart and H9c2 cardiomyoblasts, NIX primarily mediates mitophagy, though BNIP3 has the potential to compensate to a degree.

What then is the function of this mitophagy? A large body of evidence suggests that mitophagy plays a key role in removing damaged mitochondria. However, it may also play a role in metabolic remodelling, which is required during differentiation [4, 5]. It is this latter pathway that we speculate is occurring here. Metabolic reprogramming is a common phenomenon during developmental events and cell differentiation, and mitochondrial remodelling is key to drive such transition. For example, in the perinatal heart, the prime nutrient source utilised switches from carbohydrates to fatty acids and metabolism transits from glycolysis to β-oxidation for ATP production, which requires mitochondrial reprogramming [40]. PINK1-dependent mitophagy has been linked to this reprogramming and mice expressing a cardiac specific mitofusin-2 mutation, which cannot be phosphorylated by PINK1, developed progressive cardiomyopathy and died by 7-8 weeks of age [16]. NIX-mediated mitophagy has also been linked to metabolic reprogramming during differentiation. As mentioned in the results, the Boya group recently found that mitophagy was essential in mediating a glycolytic metabolic shift that was required for retinal ganglion cell differentiation [32]. Additionally, during the preparation of this manuscript, work was published that demonstrated NIX-dependent mitophagy during differentiation of adult cardiac progenitor cells [17]. In the latter study, mitophagy was shown not be essential for cardiac marker gene expression or metabolic reprogramming, but instead was suggested to be important for maintaining mitochondrial network integrity. Though we did not find a significant disruption to mitochondrial network morphology, in a somewhat similar observation, we found that loss of mitophagy did not hinder differentiation *per se*, at least in terms of marker gene expression. However, in contrast, we found that enhancing mitophagy also enhanced marker gene expression and rate-limiting glycolytic enzymes **(Fig. 7)**. Though we can only speculate at this stage, we propose that programmed mitophagy may play multiple roles during differentiation. One of these roles is likely to maintain or enhance the glycolytic status of the cell, which can produce a Warburg-like effect to aid in the production of biosynthetic molecules. How mitophagy does this is currently unknown; it is possible that it functions in a positive feedback loop with HIF1α, which we found not only to be essential for differentiation and mitophagy, but also has a well characterized role in the transcriptional upregulation of glycolysis [41, 42].

In conclusion, our findings presented in this study have expanded our understanding of the factors required for mitophagy initiation downstream of iron chelation. We propose that HIF1α is a “master regulator” of DFP-induced (and hypoxia-induced) mitophagy that controls mitochondrial tagging via upregulation of BNIP3 and NIX, while concurrently adjusting cellular metabolic activity. More importantly, our study shows that this pathway has physiological relevance and operates during cardiomyoblast differentiation.

## MATERIALS AND METHODS

### Materials

Baf A1 was purchased from Enzo Life Sciences. The following primary antibodies were used: from Cell Signalling Technology, LC3 (4108), BNIP3 (3769), NIX (12396), HSP60 (4870), PDH (3205), HK2 (2867), caspase 3 (9665), cleaved caspase 3 (9661), ULK1 (8054), phospho-ULK1 S757 (6888); from Abcam, ATP synthase subunit beta (ab13740); from R&D systems, HIF1α (MAB1536), MHC (MAB4470); from Thermo Scientific, cardiac Troponin T (MA5-12960); from Santa Cruz Biotechnology, LAMP1 (sc-20011); from Merck-Millipore PGC1α (ST1202), Pyruvate Kinase (ABS245); from BD Biosciences, OPA1 (612606); from Proteintech, α-tubulin (66031-1-Ig,). Sheep anti-Omi was produced by MRC-PPU Reagents and Services, University of Dundee. Secondary antibodies used were: goat anti-Rabbit IgG (H+L), HRP conjugate, goat anti-mouse IgG (H+L), HRP conjugate and rabbit anti-sheep IgG (H+L), HRP conjugate were purchased from Thermo Scientific. 3-Hydroxy-1,2-dimethyl-4(1H)-pyridone (DFP), CCCP, acetyl coenzyme A sodium salt, all-trans-Retinoic acid (ATRA), antimycin A and rotenone were purchased from Sigma-Aldrich. Oligomycin was obtained from NEB. FCCP was purchased from Abcam. Dulbecco's Modified Eagle Medium (DMEM), Ham's F-12 nutrient mix, Earls Balanced Salt Solution (EBSS) and Lipofectamine 2000 were purchased from Life Technologies. Foetal Bovine Serum (FBS) was purchased from Thermo Scientific. AZD8055 and all plasmids used in this study were generated by MRC-PPU Reagents and Services, University of Dundee, and are available at the following address https://mrcppureagents.dundee.ac.uk.

### Cell culture

SH-SY5Y cells were cultured in a 1:1 mix of DMEM/F-12 with 15% FBS, 2 mM L-glutamine and 1% streptomycin/penicillin. U2OS cells were cultured in DMEM with 10% FBS, 2 mM L-glutamine and 1% streptomycin/penicillin. Rat cardiomyoblast H9c2 cells (ECACC 88092904) were purchased from the European Collection of Authenticated Cell Cultures. H9c2 cardiomyoblasts were cultured in DMEM with 10% FBS, 2 mM L-glutamine and 1% streptomycin/penicillin. Cells were split when they reached 70-80% confluence to avoid the potential of spontaneous differentiation into cardiomyocytes. Differentiation was induced by changing the medium into DMEM with 1% FBS and 10 nM all-trans-Retinoic acid for 7 days. Medium was changed every other day. All cell lines were maintained at 37°C and under 5% CO_2_.

### Treatment and lysis

For experiments, cells were treated for 24 h with a final concentration of 1 mM DFP, dissolved in H_2_O at 95°C and passed through a 0.22 μm filter to sterilize. The following chemicals were added to cell media at final concentrations: 50 nM bafilomycin A1, 20 μM CCCP each made up in DMSO. For hypoxia treatment, cells were transferred to a Ruskinn INVIVO2 300 hypoxia hood at 37°C in 0.5 or 1% O_2_ and under 5% CO_2_ for 24 h. Cells were harvested by washing twice with phosphate-buffered saline (PBS) and scraping into ice-cold lysis buffer containing 50 mM HEPES pH7.4, 150 mM NaCl, 1 mM EDTA, 10% glycerol, 0.5% NP-40, 1 mM DTT, 1 mM PMSF, 1.15 mM sodium molybdate, 4 mM sodium tartrate, 10 mM β-glycerophosphate, 1 mM sodium fluoride, 1 mM sodium orthovanadate and 1x complete protease inhibitor cocktail (Roche). After incubation for 10 min on ice, lysates were cleared by centrifugation at 20,000 x g for 10 min at 4°C. Protein concentration was determined using Bradford protein assay.

### Immunoblotting

Cell lysates containing equal amounts of protein (25-35 μg) were resolved by SDS-PAGE and transferred to PVDF. The membrane was incubated with primary antibody overnight at 4°C, washed and subsequently incubated with HRP-conjugated secondary antibody for 1 h at room temperature. After washing signal detection was performed using ECL (GE Healthcare) and exposed to X-ray film.

### Transfections and stable line creation

Stable cell lines were generated by retroviral transduction. The cDNA of interest was inserted into a pBabe (for stable low expression) or pQXCIP (for stable high expression) vectors and co-transfected with GAG/POL and VSVG plasmids (Clontech) for retrovirus production using Lipofectamine 2000, following the manufacturer's instructions. Virus was harvested 48 h post-transfection, passed through a 0.45 μm filter and added to cells in the presence of 10 μg/ml polybrene (Sigma-Aldrich). Cells were selected with 2 μg/ml puromycin (Sigma-Aldrich) or 500 μg/ml hygromycin (Source Bioscience) 24 h after exposure to retroviral particles. A pool of transduced cells was utilized for subsequent experiments following selection.

For knockdown of endogenous HIF1α and NIX in SH-SY5Y, cells were transiently transfected with siRNA oligonucleotides (final concentration: 100 nM) in the presence of Transfectin (Bio-Rad). Cells were typically harvested 72 h post transfection. For knockdown of HIF1α, BNIP3 and NIX in H9c2 cells, cells were transiently transfected with siRNA (final concentration: 50 nM) in the presence of Transfectin at day 4 differentiation in differentiation medium. Cells were typically harvested after 7 days' differentiation.

The following siRNA oligonucleotides, from Thermo Scientific, were used in this study:

Non-targeting (NT): 5′-UGGUUUACAUGUCGACUAAUU-3′
Human HIF1α (AM51333): 5′-GGGUAAAGAACAAAACACATT-3′
Human NIX (4392421): 5′-CCAUAGCUCUCAGUCAGAATT-3′
Rat HIF1α (AM16708): 5′-GCUUGCUCAUCAGUUGCCATT-3′
Rat BNIP3 (s180177): 5′-GAAAAACUCAGAUUGGAUA-3′
Rat NIX (4390815): 5′-CAACAACAACUGCGAGGAATT-3′

For stable knockdown of BNIP3 an shRNA construct against human BNIP3 was cloned into a pSuperRetro vector and cells were transduced with retroviral particles as described above for pBabe and pQCXIP constructs. The shRNA sequence targeting BNIP3 used in this study was 5′-GGAAAGAAGTTGAAAGCAT-3′.

### Genome editing

The CRISPR/Cas9 system was used to generate HIF1α KO CRISPR cells. Plasmids used for the expression of the Cas9 D10A nuclease and paired guide RNAs (gRNAs) targeting exon 2 of the *HIF1A* gene were produced by the MRC PPU Reagents and Services, University of Dundee. The targeting gRNAs were co-transfected into cells using polyethylenimine (PEI, Sigma-Aldrich). One day post-transfection selection with 2 μg/ml puromycin was carried out and continued for a further 48 h. After selection, cells were diluted and plated to allow for the isolation of single colonies. Colonies were expanded before western blotting analysis to confirm mutation efficiency. For sequencing of HIF1α in WT and HIF1α CRISPR cells, genomic DNA was isolated and PCR-amplified to produce an amplicon of 401 bases encompassing exon 2 of the HIF1α gene. PCR products were then cloned into a pSC-B vector using the StrataClone Blunt PCR Cloning Kit (Agilent) as per the manufacturer's instructions and DNA sequencing was performed by the DNA Sequencing Service, University of Dundee. Sequencing of the genomic region of HIF1α CRISPR cells compared with the parental WT cell line indicated a deletion of 11 nucleotides at the middle of HIF1α exon 2:

**Figure:**
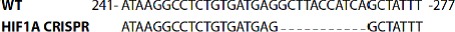


### Immunofluorescence

Cells were seeded onto sterile glass coverslips in 6-well dishes. Coverslips were washed twice with PBS, fixed with 3.7% (w/v) formaldehyde, 200 mM HEPES pH 7.0 for 10 min, washed twice with and incubated for 10 min in DMEM/10 mM HEPES pH 7.0. After one wash in PBS permeabilization was carried out using 0.2% NP-40 in PBS for 4 min. Samples were blocked by washing twice and incubation for 15 min in blocking buffer (1% (w/v) BSA/PBS). Coverslips were incubated for 1 h at 37°C with primary antibodies in blocking buffer and washed three times in blocking buffer. Coverslips were then incubated for 1 h at room temperature with Alexa Fluor coupled secondary antibodies (Life Technologies) in blocking buffer and washed an additional three times in blocking buffer. After submerging in ddH_2_O, cells were mounted onto glass slides using prolong gold antifade mountant with DAPI (Life Technologies) and visualized with a Nikon Eclipse Ti-S fluorescence microscope (Nikon) or a Zeiss LSM880 Airyscan Confocal Scanning microscope (ZEISS; Plan Apochromat X40 objective, NA 1.4). Images were processed using Adobe Photoshop or ZEISS Zen Software.

### Mitophagy assay

Cells stably expressing *mito*-QC mitophagy reporter system (mCherry-GFP-FIS1_101-152_) were seeded onto sterile glass coverslips in 6-well dishes. Coverslips were washed twice with PBS, fixed with 3.7% (w/v) formaldehyde, 200 mM HEPES pH 7.0 for 10 min, washed twice with and incubated for 10 min in DMEM/10 mM HEPES pH 7.0. After washing a final time in PBS, cells were mounted onto glass slides using prolong gold antifade mountant with DAPI. Images were processed using Adobe Photoshop or ZEISS Zen Software.

### Citrate synthase activity assay

Citrate synthase activity was determined by the method of Shepherd *et al.* [43]. Cell lysate was incubated with 100 mM Tris pH8.0, 0.1% Triton X-100, 0.1 mM acetyl-coenzyme A and 0.2 mM 5′-dithio-bis (2-nitrobenzoic acid). Reaction was started with 0.2 mM oxaloacetate and the reaction was measured at 405 nm for 1.5 minutes at 10 second intervals at 30°C using a VersaMAX plate reader (Molecular Devices).

### OCR Determination

Cellular OCR was measured using a Seahorse XF24 Extracellular Flux analyser (Seahorse Bioscience). H9c2 cells were plated at a density of 20,000 cell/well in 24-well Seahorse plates (Seahorse Bioscience) overnight. Cells were cultured in differentiation medium for 7 days. A Seahorse cartridge was calibrated 16 h prior to each experiment by placing 1 ml of calibrant solution into every well and incubating at 37°C in a CO_2_-free incubator. Cells were washed twice and medium was replaced with 0.22 μm-filtered unbuffered DMEM (pH 7.4) containing 2 mM L-glutamine, 10 mM sodium pyruvate, 10 mM HEPES and 10 mM glucose just prior to incubation for 1 h at 37°C in a CO_2_-free incubator. OCR was measured with successive injections of 1 μM oligomycin A, 1 μM FCCP and combination of rotenone and antimycin A (1 and 2 μM respectively) (all dissolved in unbuffered DMEM). Results were normalized to total protein determined by Bradford assay.

### RT-PCR

Total cellular RNA was isolated from H9c2 cells using a RNeasy® Micro Kit (Qiagen) according to the manufacturer's instructions. A 2.5-μg amount of total RNA was converted to complementary DNA (cDNA) by use of reverse transcriptase (Thermo) with oligo dT primer. The obtained cDNA samples were used as templates for or semi quantitative PCR. The mRNA expression of Troponin T, HIF1A, BNIP3, NIX and GAPDH were detected by PCR (2 minutes at 95°C, 30 seconds at 95 °C, 30 seconds at 50 °C, and 60 seconds at 72°C for 25 cycles, and then 72°C for 5 minutes) with the following primer sequences:

Troponin T: sense 5′-GGA AGA CTG GAG CGA AGA-3′, antisense 5′-AAG TTG GGC ATG AAG AGC-3′; HIF1A: sense 5′-GCT CAT CAG TTG CCA CTT CC-3′, antisense 5′-ACC TTC CAC GTT GCT GAC TT-3′; BNIP3, sense 5′-TGC ACT TCA GCA ATG GGA AT-3′, antisense 5′-ATG CTG AGA GTA GCT GTG CG-3′; NIX, sense 5′-CAT CCA CAA TGG AGA CAT GGA G-3′, antisense 5′-GGT GTG CTC AGT CGT TTT CC-3′; GAPDH: sense 5′-AGA ACA TCA TCC CTG CAT CCA-3′, antisense 5′-GCC TGC TTC ACC ACC TTC TTG-3′.

### Quantitation

Western blots were quantified by densitometry using ImageJ (https://imagej.nih.gov/ij/). Immunofluorescence-based mitophagy assay quantitation was performed manually using NIS-Elements software (Nikon) as we previously described [10] or by Volocity 6.3 Image Analysis Software (PerkinElmer). Within each experiment, analysis was performed on at least 3 fields or 10 fields (H9c2 cells) of view (typically >50 cells per experiment) for all conditions tested. Data were not collected with the counter blinded to the condition, except for Fig. 1A. In Fig. 1A, a threshold of 3 or more red-alone puncta per cell was applied to the data to determine the number of cells undergoing mitophagy.

### Statistics

Statistical significance was determined using unpaired Student's t-test for 2 group comparisons and one-way ANOVA with Dunnett's multiple comparison test for comparing the means of >2 groups to the control. For multiple comparisons, significance was determined by two-way ANOVA with Bonferroni post-test. The statistical significance is denoted on graphs by asterisks (*) or n.s. = not significant. All data were analysed using GraphPad Prism v6.0

## SUPPLEMENTAL MATERIAL

Click here for supplemental data file.

All supplemental data for this article are available online at http://www.cell-stress.com/researcharticles/2020a-zhao-cell-stress/.

## Abbreviations:

Baf A1: – bafilomycin A1;
CCCP: – carbonyl cyanide m-chlorophenyl hydrazine;
DFP: – deferiprone;
GFP: – green fluorescent protein;
KO: – knock out;
MHC: – myosin heavy chain;
mTOR: – mammalian target of rapamycin;
NT: – non-targeting;
OCR: – oxygen consumption rate;
OMM: – outer mitochondrial membrane;
OXPHOS: – oxidative phosphory-lation;
siRNA: – small interfering RNA;
WT: – wild type.
