# Supplementary material for: HIF1α-dependent mitophagy facilitates cardiomyoblast differentiation
Source: Cell Stress. 2020 Mar 4;4(5):99–113. doi: 10.15698/cst2020.05.220 (PMC7212530; doi:10.15698/cst2020.05.220)
Supplement: Supplementary file 1 [file ces-04-099-s01.pdf]

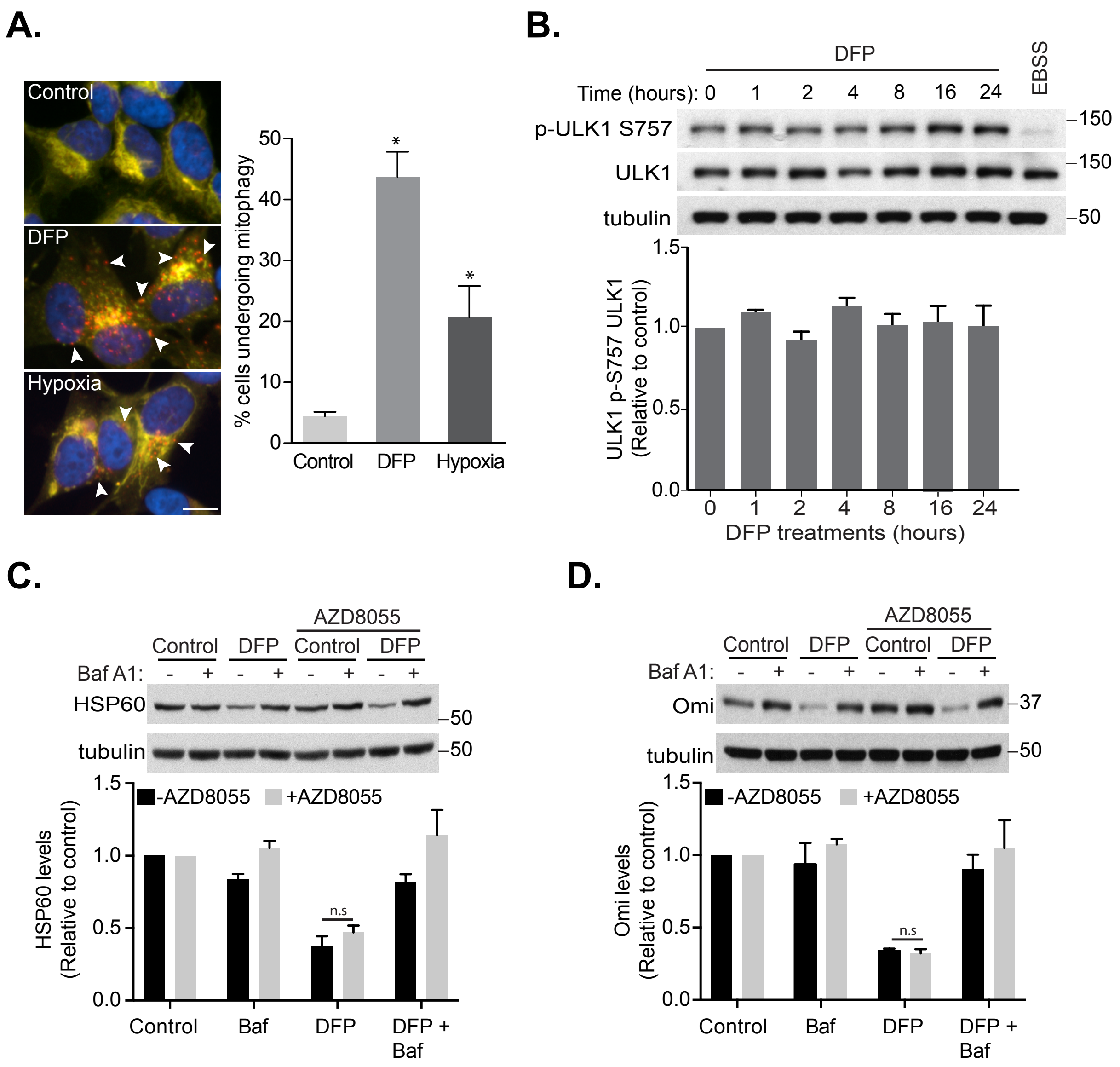

Figure S1. mTOR is not a key regulator of DFP-induced mitophagy.

(A) *mito*-QC SH-SY5Y cells were treated with 1 mM DFP or placed under hypoxic conditions (0.5% O_2) for 24 hours prior to fixation. (B) SH-SY5Y cells were treated with 1mM DFP for the indicated time or incubated with EBSS medium for 2 h. ULK1 S757 phosphorylation and ULK1 expression were examined. SH-SY5Y cells were treated with or without 1mM DFP and 1 µM AZD8055 for 24 h and 50 nM Baf A1 was added into medium for the last 16 h prior to lysis. HSP60 (C) and Omi (D) protein levels were examined. α-tubulin was used as a loading control. All quantitative data are mean ± SEM from 3 independent experiments. Scale bar, 10 µm.

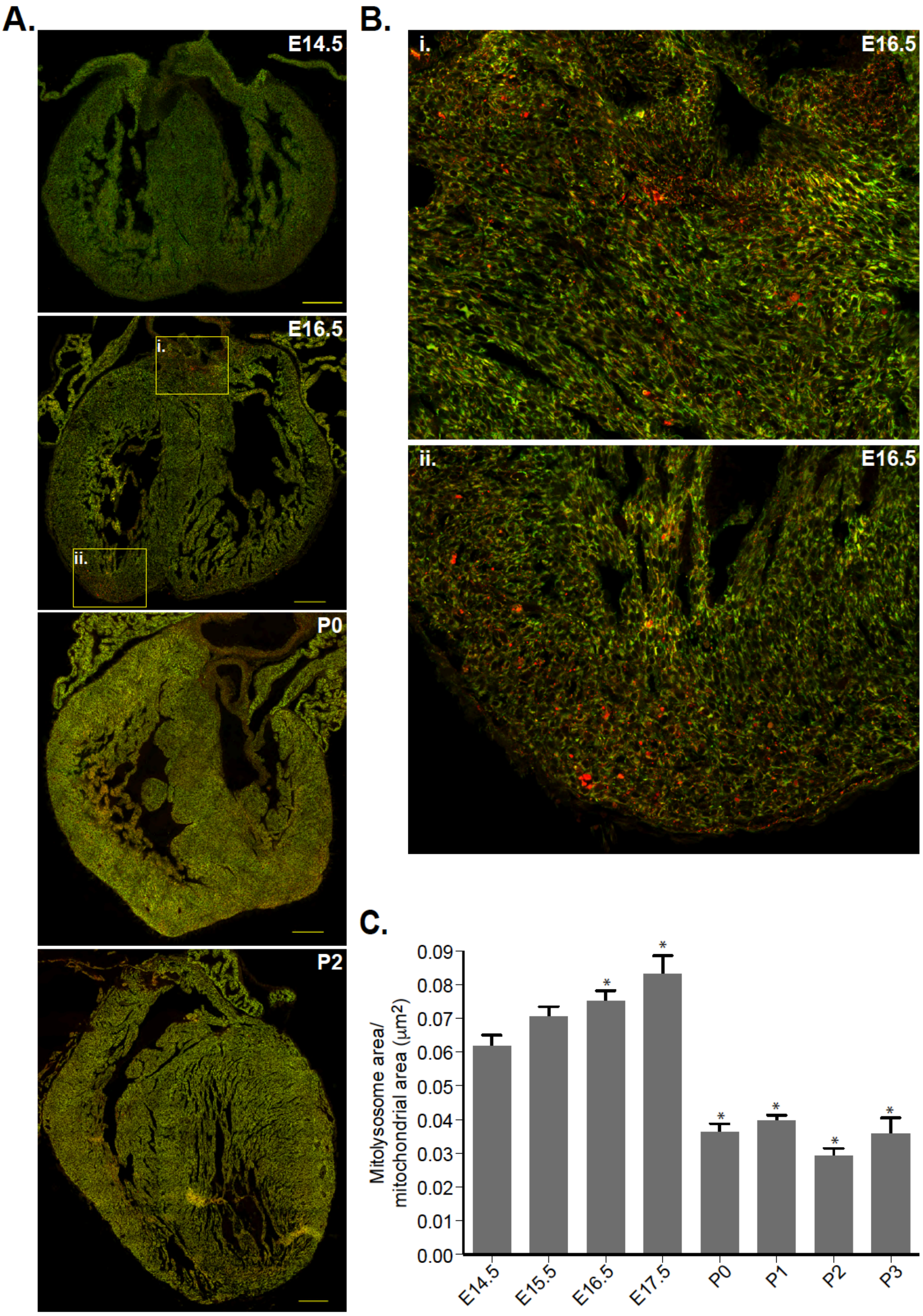

Figure S2. Mitophagy levels increase during foetal heart development. (A) Representative images of embryonic hearts from embryo 14.5 (E14.5), E16.5 and neonatal P0 and P2 of *mito*-QC reporter mice. (B) Magnified view of E16.5 heart from (A). (C) Quantitation of mitolysosomes from whole heart sections at different stages of heart development. Scale bar, 200 µm. All quantitative data are mean ± SEM from at least 5 animals per group. * $P < 0.05$.

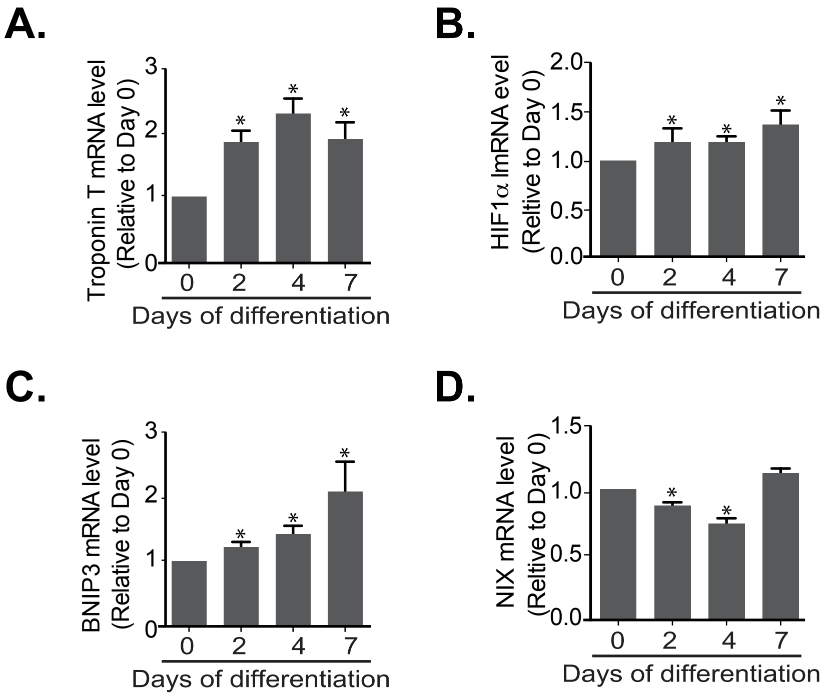

Figure S3. mRNA levels of HIF1α-related genes during cardiomyoblast differentiation. Troponin T (A) HIF1α (B), BNIP3 (C) and NIX (D) mRNA levels were examined in H9c2 cells during differentiation for 0, 2, 4 and 7 days. GAPDH was used as a loading control. All quantitative data are mean ± SEM from 3 independent experiments. * $P < 0.05$.

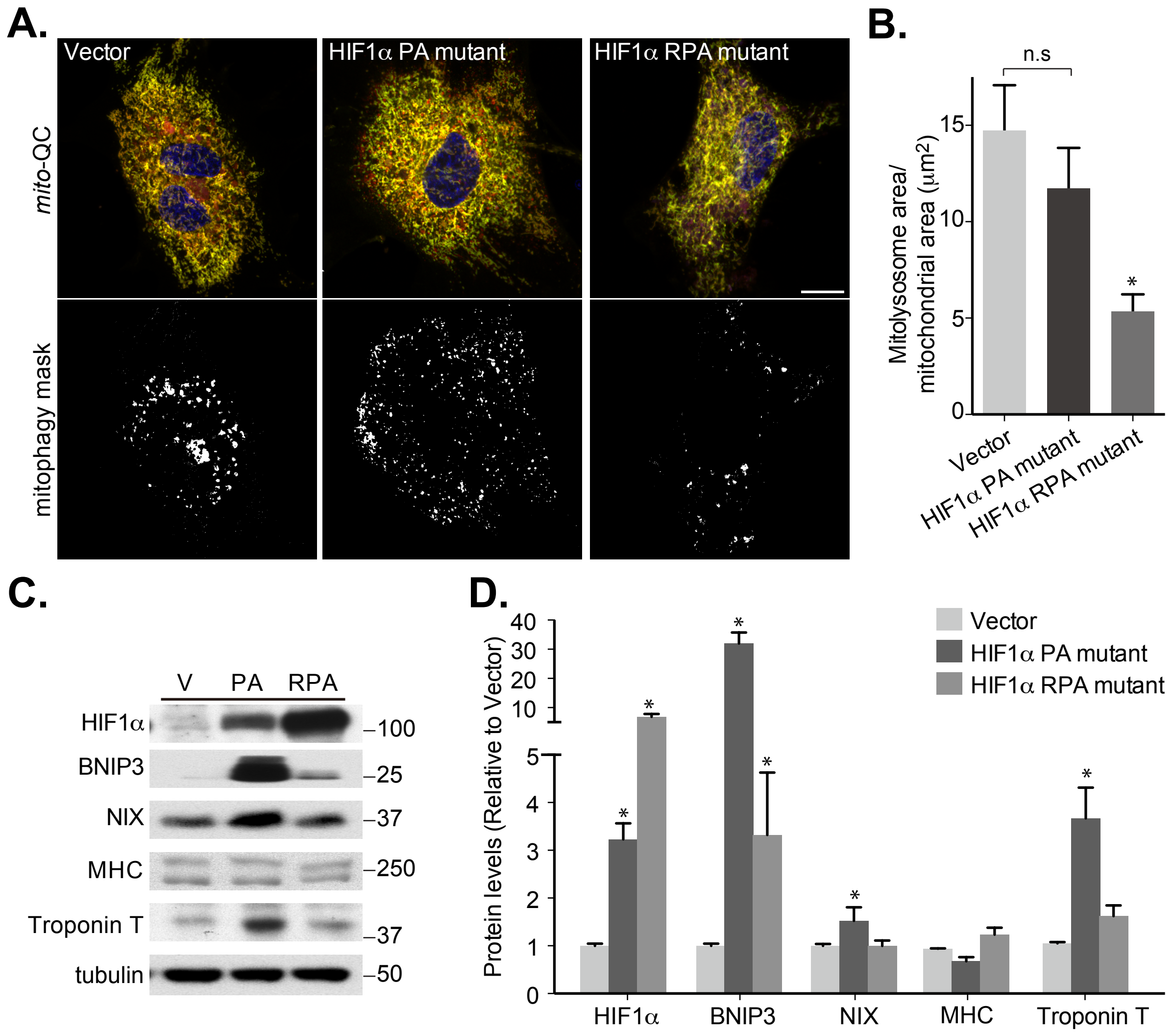

Figure S4. Increasing HIF1α stability induces cardiomyocyte differentiation. *mito*-QC H9c2 cells stably expressing either Vector, Flag-HIF1α P402A/P564A (HIF1α-PA) or Flag-HIF1α P402A/P564A/R27G (HIF1α-RPA). (A) Representative images from cells cultured in differentiation medium for 7 days. Mitophagy mask represented as the mCherry/GFP ratio intensity above the mean of mCherry intensity. (B) Quantitation from (A) of total mitolysosome area per mitochondrial content as indicated. (C-D) H9c2 cells stably expressing Vector, Flag-HIF1α-PA or Flag-HIF1α-RPA were cultured in differentiation medium for 7 days. HIF1α, BNIP3, NIX, MHC, cardiac Troponin T protein levels were examined. α-tubulin was used as a loading control. Scale bar, 20 μm. All quantitative data are mean ± SEM from 3 independent experiments. * $P < 0.05$, n.s, not significant.

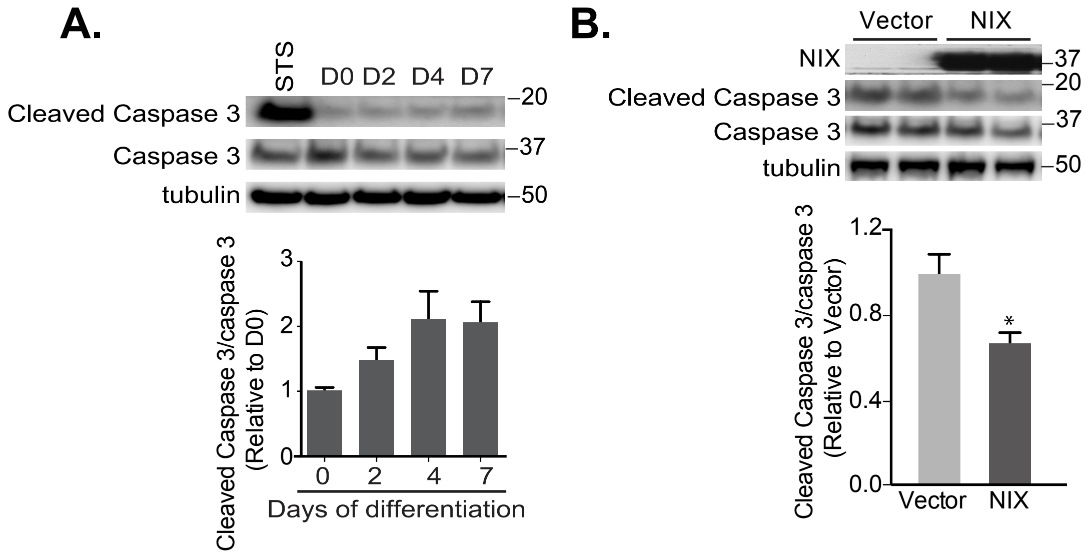

Figure S5. Overexpression of NIX does not enhance caspase cleavage during differentiation. (A) Cleaved caspase 3 and caspase 3 protein levels were examined in H9c2 cells during differentiation for 0, 2, 4 and 7 days. 1 μM Staurosporine (STS) treatment for 4 h was used as positive control. (B) H9c2 cells stably overexpressing vector or NIX cultured in differentiation medium for 7 days. NIX, cleaved caspase 3 and caspase 3 protein levels were examined. α-tubulin was used as a loading control. All quantitative data are mean ± SEM from 3 independent experiments. * $P < 0.05$.
